# CyberKnife^® ^radiosurgery in the treatment of complex skull base tumors: analysis of treatment planning parameters

**DOI:** 10.1186/1748-717X-1-46

**Published:** 2006-12-16

**Authors:** Sean P Collins, Nicholas D Coppa, Ying Zhang, Brian T Collins, Donald A McRae, Walter C Jean

**Affiliations:** 1 Department of Neurosurgery, Georgetown University Hospital, USA; 2 Department of Radiation Oncology, Georgetown University Hospital, USA; 3 Biostatistics Unit, Lombardi Comprehensive Cancer Center, Georgetown University Medical Center, USA

## Abstract

**Background:**

Tumors of the skull base pose unique challenges to radiosurgical treatment because of their irregular shapes, proximity to critical structures and variable tumor volumes. In this study, we investigate whether acceptable treatment plans with excellent conformity and homogeneity can be generated for complex skull base tumors using the Cyberknife^® ^radiosurgical system.

**Methods:**

At Georgetown University Hospital from March 2002 through May 2005, the CyberKnife^® ^was used to treat 80 patients with 82 base of skull lesions. Tumors were classified as simple or complex based on their proximity to adjacent critical structures. All planning and treatments were performed by the same radiosurgery team with the goal of minimizing dosage to adjacent critical structures and maximizing target coverage. Treatments were fractionated to allow for safer delivery of radiation to both large tumors and tumors in close proximity to critical structures.

**Results:**

The CyberKnife^® ^treatment planning system was capable of generating highly conformal and homogeneous plans for complex skull base tumors. The treatment planning parameters did not significantly vary between spherical and non-spherical target volumes. The treatment parameters obtained from the plans of the complex base of skull group, including new conformity index, homogeneity index and percentage tumor coverage, were not significantly different from those of the simple group.

**Conclusion:**

Our data indicate that CyberKnife^® ^treatment plans with excellent homogeneity, conformity and percent target coverage can be obtained for complex skull base tumors. Longer follow-up will be required to determine the safety and efficacy of fractionated treatment of these lesions with this radiosurgical system.

## Background

Lesions of the base of skull are typically slow growing, but potentially morbid tumors [1]. They rarely metastasize making local control the primary determinant of long-term survival [2]. Although surgical resection may still be the treatment "gold-standard" [3,4], radiosurgery is an appropriate treatment option for many patients [5]. However, single-fraction radiosurgical treatment may be difficult because of the potentially large size and irregular shapes of these tumors. Their proximity to critical structures also leads to a risk of radiation-induced, long-term, neurological complication [6].

The CyberKnife^® ^is a newly FDA approved radiosurgical devise for the treatment of brain lesions. Unlike the LINAC and Gamma Knife, the CyberKnife^® ^is an image-guided, frameless radiosurgery system. Treatment is delivered by a linear accelerator mounted on a flexible robotic arm. Several-hundred treatment beams are chosen out of a repertoire of greater than one thousand possible beam directions using inverse treatment planning. These beams are delivered in a non-isocentric manner via circular collimators of varying size without intensity modulation. Non-isocentric treatment allows for simultaneous irradiation of multiple lesions. The lack of a requirement for the use of a head-frame allows for staged treatment. Since the planning system has access to a large number of potential non-isocentric beams, the CyberKnife^® ^should theoretically be able to deliver a highly conformal, uniform dose with steep dose gradients [7]. Therefore, treatment with the CyberKnife^® ^radiosurgical system should minimize toxicity to surrounding structures. When compared to commonly used radiosurgical devices, such as the Gamma Knife, linear-accelerator based stereotactic radiosurgery systems with multiple arcs (LINAC), or intensity modulated radiation therapy, dosimetric studies of ellipsoid phantoms have shown that the CyberKnife^® ^radiosurgical system has the best homogeneity within the target volume and comparable conformity [8].

A dose-volume histogram (DVH) is the tool most commonly used to compare radiosurgical plans. Unfortunately, the large volume of data in these histograms does not allow for simple differentiation between multiple plans and systems [9,10]. Thus, an effort has been made to determine simple measurements for plan optimization. A conformity index is a single measure of how well the treatment dose distribution of a specific radiation treatment plan conforms to the size and shape of the target volume. In general, the conformity index of a given radiosurgical plan is dependent on target shape [11], target volume [9], collimator size [12], type of collimation (circular vs multileaf) and radiosurgical delivery system.

The new conformity index (NCI) and homogeneity index (HI) allow for the quick and simple comparison of different radiosurgical treatment plans, whether within the same radiosurgical system, or across diverse systems such as between the LINAC and Gamma Knife [13]. Conformity indices have been reported in the literature, ranging from 1.0 to 3.0 for varying radiosurgical systems [14-18]. Typically, multiple iso-center plans generated with the Gamma Knife have homogeneity indices (HI) of 2.0 to 3.0 while the LINAC plans generate homogeneity indices (HI) of 1.0 to 1.2 [17]. The significance of these differences between systems is controversial.

We determined the NCI and HI for the first 82 base of skull lesions treated at Georgetown University Hospital using the CyberKnife^® ^radiosurgical system (Accuray, Sunnyvale, CA). We undertook this study to determine the effect of target shape, target volume and proximity to critical structures on radiosurgical treatment parameters. This is the first study that we are aware of that investigates these parameters in patients treated with the CyberKnife^® ^radiosurgery system.

## Patients and methods

### Patient population

We performed a retrospective review of 262 patients with intracranial tumors, who were treated with CyberKnife^® ^stereotactic radiosurgery at Georgetown University Hospital between March 2002 and May 2005. Eighty-one patients were classified to have tumors of the skull base resulting in a total of 84 treated lesions. Thirty-three percent of these lesions had been previously irradiated. One patient was excluded from analysis because two tumor volumes were treated simultaneously making it impossible to calculate indices for each individual lesion.

Of the remaining lesions, 46 were categorized into the complex, skull base tumor group. A complex skull base tumor was defined as one that completely encircles, partially circumscribes, or directly contacts the brainstem, optic chiasm, hypophysis, or cranial nerves with meaningful remaining function. This complex tumor group consisted of 18 men and 26 women, with a median age of 53 (range: 29 – 88). These tumors were further categorized by histopathology as follows: 21 meningiomas, 6 metastatic tumors, 8 schwannomas, 7 pituitary adenomas, 1 chordoma, 2 sarcomas, and 1 glioma. The median tumor size was 7.27 cc (range: 0.62 – 98.3 cc) (Table 1 &2).

**Table 1:** Patient Characteristics

	**Control Group I (simple)**	**Control Group II (metastases)**	**Study Group (complex)**
Number of Patients	36	43	44
Number of Lesions	36	43	46
Male	16	23	18
Female	20	20	26
Age			
Min	17	21	29
Max	81	85	88
Mean	53	57	55
Median	55	58	53

**Table 2:** Skull Base Tumor Characteristics

	**Control Group I (simple) (n = 36)**	**Control Group II (metastases) (n = 43)**	**Study Group (complex) (n = 46)**
Volume (cc)			
Min	0.19	0.12	0.62
Max	206.3	66	98.3
Mean	45.61	4.87	12.6
Median	8.83	1.43	7.27

Histology			
Carcinomas	13	33	4
Chordoma	1	0	1
Gliomas	0	0	1
Malignant Gliomas	2	0	0
Melanoma	0	10	2
Meningioma	5	0	21
Pituitary Adenoma	3	0	7
Sarcomas	2	0	2
Schwannoma (not VIII)	0	0	4
Vestibular Schwannoma	10	0	4

Location			
Cavernous Sinus	2	0	15
CP Angle/IAC	12	0	6
Foramen Magnum	0	0	4
Nasopharynx	4	0	0
Orbital Apex/Parasellar	3	0	5
Paranasal Sinus	4	0	0
Petroclival	3	0	7
Sellar	3	0	7
Cerebral Hemishpere	1	34	0
Thalamus/Hypothalamus	0	2	1
Cerebellum	0	7	0
Other*	4	0	1

* Pons, mandible, infratemporal fossa

The data from the group with complex skull base tumors were compared with data from two control groups. The first group consisted of 36 patients with skull base tumors that were classified as simple. Although still located in the region of the skull base, tumors in this group had at least a 2 mm separation from the nearest critical structure. This group consisted of 16 men and 20 women, with a median age of 55 (range: 17 – 18). These tumors were also categorized by histopathology as follows: 5 meningiomas, 13 metastatic tumors, 10 schwannomas, 3 pituitary adenomas, 1 chordoma, 2 sarcomas, and 2 malignant gliomas. The median tumor size in this group was 8.83 cc (range: 0.19 – 206.3 cc) (Table 1 &2).

A second control group used for comparison consisted of 43 patients with metastatic tumors of the cerebral and cerebellar hemispheres. These lesions represented volumes that were spherical, with smooth borders, and relatively distant from critical neurovascular structures. This group consisted of 23 men and 20 women, with a median age of 58 (range: 21 – 85). These tumors were further categorized by histopatholgy as 33 metastatic carcinomas and 10 melanomas. The median tumor size in this group was 1.43 cc (range: 0.12 – 66 cc) (Table 1 &2).

### Radiosurgical treatment planning

The basic technical aspects of CyberKnife^® ^radiosurgery for cranial tumors have been described in detail (CyberKnife^® ^Radiosurgery, A Practical Guide). Briefly, the patient was placed in a supine position on a vacuum bag and a malleable thermoplastic mask was molded to the head and attached to the head support. Thin-sliced (1.25 mm) high-resolution CT images were obtained through the region of interest with the patient in the treatment position. Target volumes and critical structures were delineated by the treating neurosurgeon. The treating neurosurgeon and radiation oncologist determined the minimal tumor margin dose of the target volume and the treatment isodose. This discussion was influenced by various factors, including previous radiation to the area, tumor volume, and extent of contact and compression of critical neurological structures. In most cases, the dose was prescribed to the isodose surface that encompassed the margin of the tumor. Twelve collimator sizes are available with the CyberKnife^® ^radiosurgical system ranging from 5 mm to 60 mm. In general, a collimator size less than the maximum length of the prescribed target volume (PTV) was chosen for treatment planning [12]. An inverse planning method with non-isocenteric technique was used for all cases. The treating physician and physicist input the specific treatment criteria, limiting the maximum dose to structures such as the optic chiasm and brainstem. The majority of the treatments were given in five fractions. In general, for non-previously treated cases, treatment plans were deemed acceptable if the maximum dose to critical structures was less than 2000 cGy in five fractions. Non-anatomical dose constraint structures were commonly incorporated to aid the optimization process in minimizing the dose to critical structures. The planning software calculated the optimal solution for treatment. The DVH of each plan was evaluated until an acceptable plan was generated.

### Treatment planning parameters

#### Target volume

Target volume was defined as the volume contoured on the planning CT scan by the treating neurosurgeon. No margin was added to the target volume. In this study, there was no limit set on the treatable target volumes.

#### Homogeneity Index

The homogeneity index (HI) describes the uniformity of dose within a treated target volume, and is directly calculated from the prescription isodose line chosen to cover the margin of the tumor:

HI=(maximum dose)(prescription dose)
 MathType@MTEF@5@5@+=feaafiart1ev1aaatCvAUfKttLearuWrP9MDH5MBPbIqV92AaeXatLxBI9gBaebbnrfifHhDYfgasaacH8akY=wiFfYdH8Gipec8Eeeu0xXdbba9frFj0=OqFfea0dXdd9vqai=hGuQ8kuc9pgc9s8qqaq=dirpe0xb9q8qiLsFr0=vr0=vr0dc8meaabaqaciaacaGaaeqabaqabeGadaaakeaacqqGibascqqGjbqscqGH9aqpdaWcaaqaaiabcIcaOiabb2gaTjabbggaHjabbIha4jabbMgaPjabb2gaTjabbwha1jabb2gaTjabbccaGiabbsgaKjabb+gaVjabbohaZjabbwgaLjabcMcaPaqaaiabcIcaOiabbchaWjabbkhaYjabbwgaLjabbohaZjabbogaJjabbkhaYjabbMgaPjabbchaWjabbsha0jabbMgaPjabb+gaVjabb6gaUjabbccaGiabbsgaKjabb+gaVjabbohaZjabbwgaLjabcMcaPaaaaaa@5A0D@

#### New Conformity Index

The new conformity index (NCI) as formulated by Paddick [13], and modified by Nakamura [16] describes the degree to which the prescribed isodose volume conforms to the shape and size of the target volume. It also takes into account avoidance of surrounding normal tissue.

NCI=[(treatment volume)×(prescription isodose volume)](volume of the target covered by the prescription isodose volume)2
 MathType@MTEF@5@5@+=feaafiart1ev1aaatCvAUfKttLearuWrP9MDH5MBPbIqV92AaeXatLxBI9gBaebbnrfifHhDYfgasaacH8akY=wiFfYdH8Gipec8Eeeu0xXdbba9frFj0=OqFfea0dXdd9vqai=hGuQ8kuc9pgc9s8qqaq=dirpe0xb9q8qiLsFr0=vr0=vr0dc8meaabaqaciaacaGaaeqabaqabeGadaaakeaacqqGobGtcqqGdbWqcqqGjbqscqGH9aqpdaWcaaqaaiabcUfaBjabcIcaOiabbsha0jabbkhaYjabbwgaLjabbggaHjabbsha0jabb2gaTjabbwgaLjabb6gaUjabbsha0jabbccaGiabbAha2jabb+gaVjabbYgaSjabbwha1jabb2gaTjabbwgaLjabcMcaPiabgEna0kabcIcaOiabbchaWjabbkhaYjabbwgaLjabbohaZjabbogaJjabbkhaYjabbMgaPjabbchaWjabbsha0jabbMgaPjabb+gaVjabb6gaUjabbccaGiabbMgaPjabbohaZjabb+gaVjabbsgaKjabb+gaVjabbohaZjabbwgaLjabbccaGiabbAha2jabb+gaVjabbYgaSjabbwha1jabb2gaTjabbwgaLjabcMcaPiabc2faDbqaaiabcIcaOiabbAha2jabb+gaVjabbYgaSjabbwha1jabb2gaTjabbwgaLjabbccaGiabb+gaVjabbAgaMjabbccaGiabbsha0jabbIgaOjabbwgaLjabbccaGiabbsha0jabbggaHjabbkhaYjabbEgaNjabbwgaLjabbsha0jabbccaGiabbogaJjabb+gaVjabbAha2jabbwgaLjabbkhaYjabbwgaLjabbsgaKjabbccaGiabbkgaIjabbMha5jabbccaGiabbsha0jabbIgaOjabbwgaLjabbccaGiabbchaWjabbkhaYjabbwgaLjabbohaZjabbogaJjabbkhaYjabbMgaPjabbchaWjabbsha0jabbMgaPjabb+gaVjabb6gaUjabbccaGiabbMgaPjabbohaZjabb+gaVjabbsgaKjabb+gaVjabbohaZjabbwgaLjabbccaGiabbAha2jabb+gaVjabbYgaSjabbwha1jabb2gaTjabbwgaLjabcMcaPmaaCaaaleqabaGaeGOmaidaaaaaaaa@C6C7@

#### Percent Target Coverage

PTC = The percentage of the target volume covered by the prescription isodose.

### Radiosurgical treatment delivery

Image-guided radiosurgery was employed to eliminate the need for stereotactic frame fixation. Using computed tomography planning, target volume locations were related to radiographic landmarks of the cranium. With the assumption that the target position is fixed within the cranium, cranial tracking allows for anatomy based tracking relatively independent of patient's daily setup. Position verification was validated several times per minute during treatment using paired, orthogonal, x-ray images.

### Statistical analysis

Chi-square test or two-sample t-test was used to test the distributions of the characteristics between the simple and complex groups. To assess the association between radiation treatment parameters and the tumor volume, simple linear regressions on tumor volume for each of the three indices were performed. The estimates of the slopes and their 95% confidence intervals were determined. Pearson's correlation coefficients and their 95% confidence intervals were calculated for the whole cohort.

## Results

### Patient and tumor characteristics

The characteristics of the two treatment groups including their gender, age, tumor histology and locations are detailed below and summarized in Tables 1 and 2. The simple group was composed predominantly of malignant lesions and vestibular schwannomas, while the complex group consisted primarily of cavernous sinus meningiomas and pituitary adenomas.

### Overall radiosurgical parameters: effect of tumor shape

Overall, compared to previously reported conformity indices for LINAC and GammaKnife systems, the CyberKnife^® ^radiosurgical system compared favorably with a mean NCI of 1.6–1.8 and a mean HI of 1.2–1.3 (Table 3). The standard percentage target coverage of 95% was not compromised to obtain these values.

**Table 3:** Radiosurgery Treatment Plan

	**Control Group I (simple) (n = 36)**	**Control Group II (metastases) (n = 43)**	**Study Group (complex) (n = 46)**
Dose (cGy)			
Min	900	1500	1500
Max	3500	3000	3500
Mean	2301	1905	2387
Median	2500	1900	2500

Treatment Stages			
Min	3	1	1
Max	10	5	5
Mean	5.2	1.5	4.7
Median	5	1	5

Homogeneity Index			
Min	1.11	1.11	1.07
Max	1.49	1.54	1.67
Mean	1.26	1.21	1.24
Median	1.25	1.19	1.25

New Conformity Index			
Min	1.04	1.04	1.27
Max	2.59	3.11	2.27
Mean	1.66	1.73	1.67
Median	1.57	1.64	1.57

Percent Target Coverage (%)			
Min	82.5	79.6	80.2
Max	99.9	100.0	99.9
Mean	95.9	97.0	94.3
Median	97.5	99.1	94.7

Base of skull lesions commonly have irregular, non-spherical shapes due to the presence of dural tails and the anatomy of the region. To determine the effect of tumor shape on radiosurgical parameters, a group of spherical cerebellar and cerebral hemisphere metastases were analyzed for comparison (Control Group II (metastases)). The calculated indices for this group were similar to the indices obtained for the base of skull lesions: mean NCI of 1.73 and a mean HI of 1.21 (Table 3). These data suggest that the CyberKnife^® ^radiosurgical system generates conformal and homogeneous plans independent of tumor shape.

### Comparison of radiosurgical parameters between complex and simple base of skull lesions

**Figure 1:**
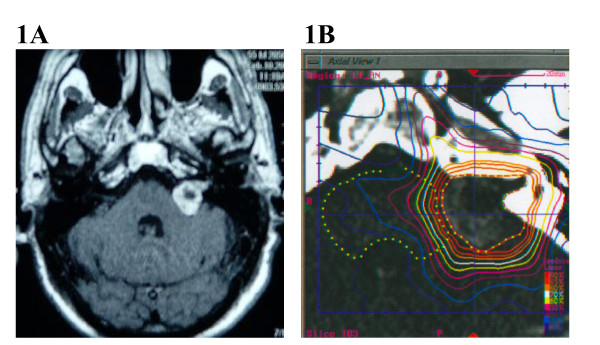
(A) A 51 year old woman presented with progressive hearing loss. An axial MRI of the brain After gadolinium administration demonstrated a left cerebellopontine angle acoustic neuroma. (B) Planning CT scan with IV contrast. The patient was treated with 2500 cGy to the 79% isodose line in five stages.

**Figure 2:**
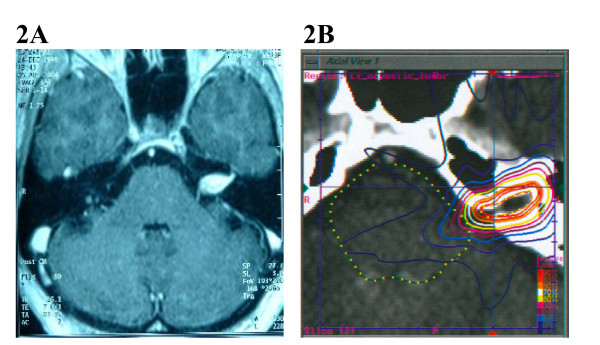
(A) A 77 year old woman presented ten years after craniotomy for acoustic neuroma resection with deafness. An axial MRI of the brain after gadolinium administration demonstrated radiographic progression of disease within the left internal acoustic meatus. (B) Planning CT scan with IV contrast. The patient was treated with 2500 cGy to the 84% isodose line in five stages.

Complex base of skull lesions were defined as one that completely encircles, partially circumscribes, or directly contacts the brainstem, optic chiasm, hypophysis, or cranial nerves with meaningful remaining function (see Figure 1 for example). All other lesions were classified as simple base of skull lesions (see Figure 2 for example). Table 4 gives the distribution of tumor volume, homogeneity index, new conformity index, and percentage target coverage for the simple and complex groups, respectively.

**Table 4:** Statistical Analysis

	**Control Group I (simple) (n = 36)**	**Study Group (complex) (n = 46)**	**Difference of the means (95% CI)**	**p Value**
Volume (cc)				
Mean	45.61	12.60	---	0.0059^a^
Median	8.83	7.27		

Homogeneity Index				
Mean	1.26	1.24	0.019 (-0.024, 0.063)	0.38^a^
Median	1.25	1.25		

New Conformity Index				
Mean	1.66	1.67	-0.007 (-0.155, 0.142)	0.93^a^
Median	1.57	1.57		

Percent Target Coverage (%)				
Mean	95.9	94.3	1.581 (-0.304, 3.466)	0.10^b^
Median	97.5	94.7		

a = t-test

b = t-test for log transformed percent target coverage

Overall, there is no statistically significant difference in homogeneity index, new conformity index and percentage target coverage between the two groups at the 5% level. There was a trend towards lower percent target coverage in the complex group, however this was not statistically significant. These data suggest that the CyberKnife^® ^radiosurgical system generates acceptable plans independent of the proximity of adjacent critical structures to the target volume.

### Relationship between tumor volume and radiosurgical parameters

**Figure 3:**
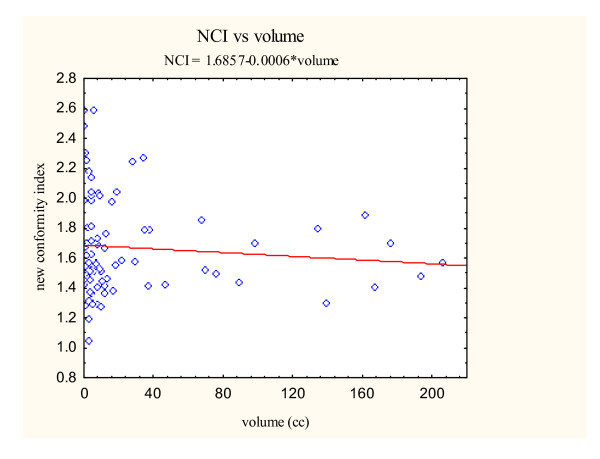
New conformity index versus volume scatter plot with correlation analysis.

**Figure 4:**
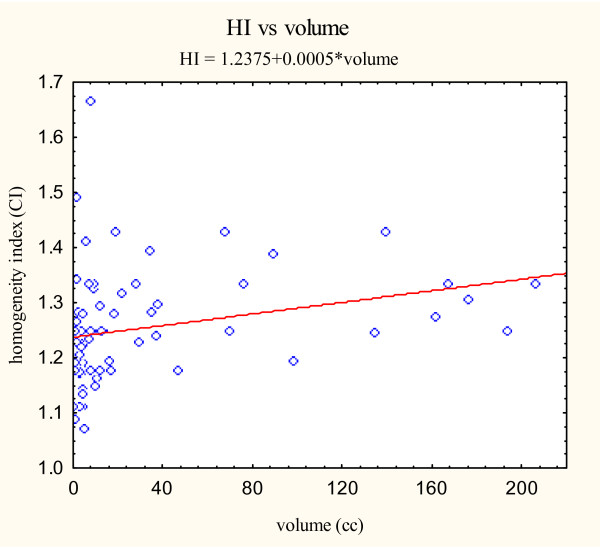
Homogeneity index versus volume scatter plot with correlation analysis.

**Figure 5:**
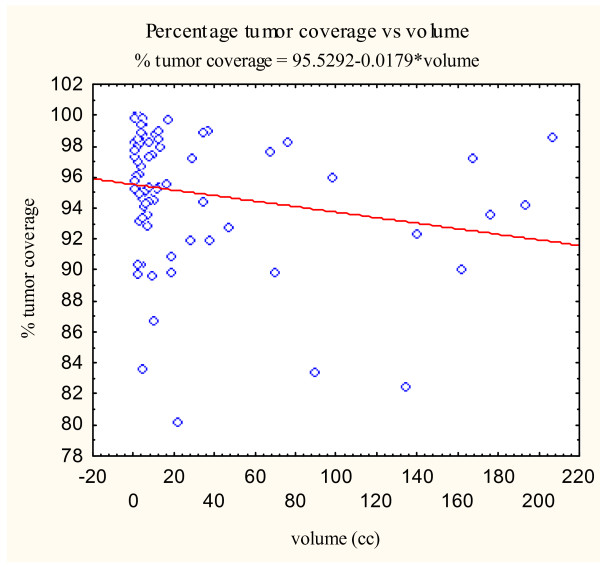
Percent target coverage versus volume scatter plot with correlation analysis.

Previous radiosurgical series have shown that radiosurgical indices can be influenced by target volume [9]. In our study, the mean tumor volumes differed significantly between the simple and complex groups (p = 0.0059) (Table 4). For the simple group, the mean tumor volume was 45.6 cm^3^. The mean tumor volume for the complex group was smaller at 12.5 cm^3^. Hence, we explored the relationship between target volume and radiosurgical indices using the CyberKnife^® ^treatment planning system. To assess the association between the three radiosurgical treatment parameters (new conformity index, homogeneity index, and percentage of tumor coverage) and the target volume, scatterplots were constructed from the data obtained from all skull base tumors (Figure 3, 4, 5).

Simple linear regressions on the tumor volume for each of the three indices were then performed. The estimates of the slopes are given in Table 5. The estimated slopes for all indices are near zero. Pearson's correlation coefficients were also calculated as seen in Table 5. All Pearson correlation coefficients were less than ± 0.4 suggesting a poor correlation between the examined variables. Therefore, tumor volume does not appear to markedly effect radiosurgical parameters when using the CyberKnife^® ^radiosurgical treatment planning system in our patient population.

**Table 5:** Linear Regression Analysis: Radiosurgical Indices as a Function of Lesion Volume

	**y-intercept**	**Slope**	**Pearson's Correlation Coefficient**
Homogeneity Index	1.2377	0.00054	0.3715
New Conformity Index	1.6995	-0.00076	-0.1365
Percent Target Coverage (%)	95.52	-0.00030	-0.3875

## Discussion

The CyberKnife^® ^radiosurgical system has several advantages over conventional radiosurgical systems. Cranial tracking, using skeletal anatomy to position the radiation beam, is as precise as frame-based approaches and eliminates the need for headframes [19]. In phantom studies, the system's precision has been shown to compare favorably to frame-based systems [20]. Its sub-millimeter clinical accuracy is due both to improvements in radiation delivery and target localization [21,22]. In addition, most LINAC and Gamma Knife systems use forward planning with user-selected arcs and beams. The CyberKnife^® ^radiosurgical system employs inverse planning algorithms based on specific constraints to critical structures. In theory, inverse planning should allow for easily obtainable, optimized plans. The appropriate measure(s) of plan optimization is still debated [9].

Assessment of success in radiosurgery requires time for data to mature. But treatment-planning parameters, including conformity and homgeneity, can be assessed much earlier. In this study, we demonstrate that the CyberKnife^® ^radiosurgical system generates plans with excellent conformity and homogeneity. Theoretically, improvements in conformity should improve local control and decrease complications in the treatment of skull base lesions with adjacent critical structures. These general principles have found acceptance in the treatment of other sites with radiation therapy [23,24].

When irradiating complex skull base tumors that abut or displace critical normal structures the dose constraints to those normal structures may cause areas of under-dosing within the target volume. Of particular concern is that the resulting low dose regions within the tumor volume will increase the rate of local failure. In two radiosurgical series, the majority of local failures were due to tumor progression just outside the prescribed isodose volume [25,26]. At least one report in the literature has documented that increased conformity is paradoxically associated with poorer outcomes [27]. It has been suggested that improved conformity may lead to underdosing microscopic disease, not visible with current imaging modalities. However, in the study cited above, the poorer outcomes were likely due to the fact that conformity improves with increasing size of the lesion and is not related to an intrinsic and pure relationship between conformity and outcome. As logic dictates, increased rate of local failure is predicted to be dependent on both the dose minimum and the volume of this dose. Currently, percent target coverage is used as a surrogate for quantifying these low dose areas. In this study, percent target coverage was maintained across all groups. Longer follow-up is required to judge the effectiveness of this system in terms of local tumor control.

Dose homogeneity is a second measure by which radiosurgical plans are compared. The homogeneity index (HI), the maximum dose within the target volume divided by the prescription isodose (MDPD), is a commonly used measure of dose homogeneity. The importance of dose homogeneity in radiosurgical outcomes is controversial. Inhomogeneous high central doses achieved with some radiosurgical treatment systems may provide improved local control [28]; however, this increased local control may come with an increased risk of neurologic complications [29]. A homogeneity index of less than 2.0 is felt to balance the risk of local failure and neurologic injury (RTOG guidelines) [28]. Homogeneity indices less than 2.0 are especially important in treating large tumors or tumors in close proximity to critical structures [29]. Even though we did not place limitations on target volume or proximity of critical structures, we were able to obtain homogeneity indices less than 2.0 for every plan. Homogeneity of dose distributions for the CyberKnife^® ^was favorable compared with devices using multiple isocenters which are typically 2.0. In the opinion of the authors, allowable target volumes and proximity to critical structures need to be determined in the context of the homogeneity index. Larger target volumes and smaller separation from critical structures may be acceptable for systems that consistently generate low homogeneity indices [5].

## Abbreviations

FDA, Federal Drug Administration; LINAC, Linear Accelerator; DVH, Dose Volume Histogram; NCI, New Conformity Index; HI, Homogeneity Index; PTV, Planning Treatment Volume; PTC, Percent Target Coverage; MRI, Magnetic Resonance Imaging; CT, Computed Tomography.

## Competing interests

The author(s) declare that they have no competing interests.

## Authors' contributions

**SC**: Drafted the manuscript and participated in data analysis, prepared the manuscript for submission, created tables and results section

**NC**: Drafted the manuscript and participated in data analysis, prepared the manuscript for submission, created tables and results section

**YZ**: Biostatistical analysis

**BC**: Participated in treatment planning and manuscript revision

**DM**: Extracted data from treatment planning systems; manuscript revision

**WJ**: Participated in treatment planning and manuscript revision; corresponding author
